# Applications of Supercritical Fluid Extraction (SFE) of Palm Oil and Oil from Natural Sources

**DOI:** 10.3390/molecules17021764

**Published:** 2012-02-10

**Authors:** Mohammed Jahurul Haque Akanda, Mohammed Zaidul Islam Sarker, Sahena Ferdosh, Mohd Yazid Abdul Manap, Nik Norulaini Nik Ab Rahman, Mohd Omar Ab Kadir

**Affiliations:** 1 School of Industrial Technology, University Sains Malaysia, Minden, Penang 11800, Malaysia; Email: jahurulhaque@yahoo.com (M.J.H.A.); sahenaferdosh@gmail.com (S.F.); 2 Department of Pharmaceutical Technology, Kulliyyah of Pharmacy, International Islamic University, Kuantan Campus, Kuantan, Pahang D/M 25200, Malaysia; Email: zaidul@iium.edu.my; 3 Department of Food Technology, Faculty of Food Science and Technology, University Putra Malaysia, UPM Serdang, Selangor 43400, Malaysia; Email: myazid@food.upm.edu.my; 4 School of Distance Education, University Sains Malaysia, Minden, Penang 11800, Malaysia

**Keywords:** supercritical fluid extraction, palm oil, palm kernel oil, major and minor constitutes of palm oil, use of palm oil, palm oil industry, bioactive lipid components

## Abstract

Supercritical fluid extraction (SFE), which has received much interest in its use and further development for industrial applications, is a method that offers some advantages over conventional methods, especially for the palm oil industry. SC-CO_2_ refers to supercritical fluid extraction (SFE) that uses carbon dioxide (CO_2_) as a solvent which is a nontoxic, inexpensive, nonflammable, and nonpolluting supercritical fluid solvent for the extraction of natural products. Almost 100% oil can be extracted and it is regarded as safe, with organic solvent-free extracts having superior organoleptic profiles. The palm oil industry is one of the major industries in Malaysia that provides a major contribution to the national income. Malaysia is the second largest palm oil and palm kernel oil producer in the World. This paper reviews advances in applications of supercritical carbon dioxide (SC-CO_2_) extraction of oils from natural sources, in particular palm oil, minor constituents in palm oil, producing fractionated, refined, bleached, and deodorized palm oil, palm kernel oil and purified fatty acid fractions commendable for downstream uses as in toiletries and confectionaries.

## 1. Introduction

The supercritical fluid extraction (SFE) technology has advanced tremendously since its inception and is a method of choice in many food processing industries. Over the last two decades, SFE has been well received as a clean and environmentally friendly “green” processing technique and in some cases, an alternative to organic solvent-based extraction of natural products. The most recent advances of SFE applications in food science, natural products, by-product recovery, pharmaceutical and environmental sciences have been published in extensive reviews [1]. The authors of these papers also discussed comprehensively the applications of SFE to extract high value compounds from food and natural products, as well as heavy metals recovery, enantiomeric resolution or drug delivery systems and the development of new separation techniques, such as using supercritical fluids to separate components of the extract, resulting in augmented quality and purity [2]. This makes SFE a valuable technique in the extraction of natural products, including fats and oils. These advantages are being used to remove caffeine from coffee [3], and harmful components from nutraceutical products [4]. SFE extracts the oil or desired element from the subjected material in shorter time compared to the conventional methods. Supercritical fluid extracts are typically sterilized, contamination free and the valuable components remain in chemically natural state [5,6]. SFE technology has also been investigated for the degumming and bleaching of soybean oil [7], palm oil [8,9], purification of used frying oil [10]; fractionation of butter oil [11] and beef tallow [12]. 

Supercritical fluid solvents are of interest in chemical processes both for their involvement in chemical reactions as well as their solvent effects, that are influenced by pressure and temperature. Supercritical fluid (SCF) solvents such as SC-CO_2_ are intermediates between liquid and gases and considered important in the separation processes based on the physicochemical characteristics including density, viscosity, diffusivity and dielectric constant which are easily manipulated by pressure and temperature. A fluid that exists at a state above the critical temperature (T_c_) and the critical pressure (P_c_) is in a supercritical condition and the uniqueness of a SCF is that its density is pressure dependent. The density can be attuned from liquid to vapor condition with continuity. 

In a SCF extraction process, the most important regions in the pressure-temperature-composition space are those of (i) 2-phase, liquid-vapour (LV), solid-vapor (SV), or liquid-liquid (LL) equilibrium; (ii) 3-phase, liquid-liquid-vapor (LLV), solid-liquid-vapour (SLV), solid-solid-vapor (SSV) equilibria, and sometimes; (iii) 4-phase equilibria: solid-SCF mixtures. As an extractive solvent, SCF can break up a multi component mixture based on the different volatile capacities of each component. Supercritical fluid extraction facilitates the detachment of the extract from the supercritical fluid solvent by simple expansion. An added benefit is derived from the liquid like densities of the supercritical fluids with superior mass transfer distinctiveness that enables the easy release of solutes, compared to other liquid solvents. This uniqueness is owed to the high diffusion and very low surface tension of the supercritical fluid that enables easy infiltration into the permeable make-up of the solid matrix to reach the solute [13,14,15]. 

Since the early 1980s, the use of SC-CO_2_ in the extraction of oil or lipid from various sources, both plants [16,17,18] and animals [19,20] has been studied extensively. In addition, the application of SC-CO_2_ in the extraction of minor constituents from various plant sources has also been widely studied [21,22]. Recently, Pourmortazavi *et al*. [23] reported that carbon dioxide is used in more than 90% of all analytical supercritical fluid extractions. The low critical temperature of carbon dioxide (31.1 °C) makes it attractive for thermally labile food products. Other solvents including ethane and propane are also used as supercritical fluids for the extraction of natural compounds. These solvents have high solvating power enabling higher solubility of lipid components compared to SC-CO_2_. The main demerits of ethane and propane are their flammability and high cost.

Organic solvents such as hexane are used widely in lipid extraction and fractionation operations that can achieve almost complete recovery of oil from a sample matrix. In many countries, health and safety regulations are getting stricter in addressing environmental problems created by the use of organic solvents and these issues are forcing the industries to search for alternative processing methods. The solvent is unsafe to handle and unacceptable as it is harmful to human health and the environment, restricting its use in the food, cosmetic and pharmaceutical industries [24]. Furthermore, the major drawback of the solvent extracted products is the high level of residues left in the final products that must be desolventized before consumption. Therefore, SC-CO_2_ is seen as a more favorable alternative to organic solvents in the extraction of fats and oils, and meets the growing consumer demand for safe natural fats and oils of excellent quality [25].

Pressure, temperature, particle size and sample pre-treatment are most important factors in oils as well as high value bioactive desired compounds extraction from the natural sources using supercritical fluid, because of the influence they have on the quality of the extracts. Many researchers have reported in detail the influential extraction parameters such as pressure, temperature, sample particle size and pre-treatment in the literature [26,27,28,29]. Generally, the solubility of the solute in the supercritical fluid solvent depends on the choice of SFE operating pressure and temperature [30]. These extraction parameters are in fact, directly responsible for the extract composition and component functionalities [31]. The performance of SFE or the quality of extracts can also be influenced by other factors such as bed geometry, the number of extraction and separation vessels and the solvent flow rate [32]. The main aim of this review paper is to give a detailed and updated discussion and analysis on research that has been conducted on the use of SFE in the extraction of oils, with special reference to palm oil, palm kernel oil and other oils from natural sources.

## 2. Advantages and Economy of SC-CO_2_ as a Solvent

The application of carbon dioxide as a supercritical fluid has been extensively studied over the past three decades, especially in food processing. Supercritical fluid (SCF) at its critical temperature and pressure shows unique properties different from those of either gasses or liquids under standard conditions. Carbon dioxide can easily penetrate through the solid matrix and dissolve the desired extract due to its dual gaseous and liquid-like properties. SFE exploits the ability of chemicals to function as outstanding solvents for certain desired components under a suitable set of pressure and temperature conditions. The final products obtained by supercritical carbon dioxide (SC-CO_2_) extraction retain their quality and the stability of thermally labile natural components is assured without changing the bioactivity of natural molecules. SFE has been shown as a technically feasible alternative to both extraction and refining processes, especially for natural oils and bioactive compounds [33]. It is fairly rapid because of the low viscosity and high diffusivity associated with SCF. Extraction selectivity can be achieved by changing the temperature, pressure and co-solvent and the extracted material is easily recovered by simply depressurizing, allowing the supercritical CO_2_ to return to gaseous state and evaporate leaving little or no traces of solvent [34]. The natural fats and oils obtained by SC-CO_2_ extraction are of excellent quality and are comparable to those obtained by organic solvent extraction methods [35]. The solvent power of SC-CO_2_ is good since it dissolves non-polar to slightly polar compounds. The addition, of small quantities of polar organic solvent as modifiers can improve the extraction of polar compound by increasing the solubility of the analyte in CO_2_, or by reducing its interaction with the sample matrix or both [36]. Examples of the substances used thus far as supercritical solvents and their critical temperature and pressure are given in Table 1 [37]. 

**Table 1:** Examples of substances used as supercritical solvents and its corresponding critical temperature and pressure. Reproduced with substantial modification from [37].

Gases	Critical Temperature (K)	Critical Pressure (MPa)
Carbon dioxide	304.17	7.38
Ethane	305.34	4.87
Methane	190.55	4.59
Ethylene	282.35	5.04
Propane	369.85	4.24
Nitrous oxide	309.15	7.28
Acetylene	308.70	6.24
Hydrogen	33.25	1.29
Nitrogen	126.24	3.39
Oxygen	154.58	5.04
Neon	44.40	2.65
Argon	150.66	4.86
Xenon	289.70	5.87

Among them, CO_2_ is the most common supercritical fluid solvent, and has been extensively studied for its potential applications in many different fields, including the food processing industries. Due to the low critical temperature and pressure, low cost, wide availability, non-flammability and environmentally friendliness, supercritical CO_2_ is the most acceptable supercritical solvent in food applications as well as in other applications without any declaration [38]. As an example, cholesterol was shown to be more soluble in supercritical ethane than in SC-CO_2_ [29]. As ethane is much more costly than CO_2_, the use of CO_2_/ethane and CO_2_/propane mixtures can be a good alternative for removal of cholesterol from food by compromising between higher ethane cost and better cholesterol removal efficiency, so supercritical fluid extraction can reduce the extraction and separation costs. Carbon dioxide can be recycled or reused from large scale SFE processes and is environmentally safe [39]. The CO_2_ used is largely a byproduct of industrial processes or brewing, and its use as supercritical solvent does not cause any extra emissions and cost. 

## 3. Palm Oil and Palm Kernel Oil Production

Palm oil, also called palm fruit oil, is a natural edible vegetable oil obtained from the fruit of the palm tree. Based on the Hamburg-based Oil World Trade journal report, the global fats and oil production were 160 million tons in 2008. Palm oil and palm kernel oil contributed about 48 million tons or 30% of the total, while soybean oil was 37 million tons or 23%. The palm oil has surpassed soybean oil as the most widely produced vegetable oil in the World. Palm is quite unique in that it yields two types of oil: palm oil from the mesocarp and palm kernel oil from the palm kernels. Palm kernels from where palm kernel oils (PKO) are obtained are, in fact, a by-product obtained from the processing of the palm fruits and its production has also been increasing. World production of palm kernel oil was 3,236 metric tons in 2003, of which Malaysia produced 1,644 metric tons [40]. The largest portion (90%) of palm oil and its products are used for consumption, while the remaining 10% is utilized for nonedible purposes [41].

Malaysia is the second largest palm oil producer around the world, and produced 17.7 million tons of palm oil in 2008. Malaysia exports about 60% of palm oil around the World and this makes a significant contribution to its national economy [40]. Moreover, Malaysian palm oil is currently fulfilling most of the increasing global demands for oils and fats. This puts Malaysia in a favorable position to become a major supplier of raw materials for oleochemical industries both locally and overseas. Oleochemicals that are widely used in lubricants, plastics, resins, soaps, surfactants, emulsifiers, cosmetics, toiletries and other chemicals for the textile industries are produced from palm oil and its by-products. It is predicted that by 2012 palm oil will be the leading internationally traded edible oil. Palm oil is also cheaper than peanut oil, corn oil or soybean oil [42].

## 4. Major Constituents of Palm Oil and Palm Kernel Oil

Like all naturally occurring edible oils, palm oil and palm kernel oil are constituted mainly by triglycerides (TGs). More than 95% of palm oil consists of mixtures of TGs, formed from one molecule of glycerol with three fatty acids. Palm oil and palm kernel oil are high in saturated fatty acids, about 50% and 80%, respectively. The ratio of unsaturated and saturated fatty acids in palm oil is well balanced. It contains 40% monounsaturated fatty acid (oleic acid), 10% polyunsaturated fatty acid (linoleic acid), 45% palmitic acid and 5% stearic acid (saturated fatty acids) [43]. The metabolites due to the biosynthesis of triglycerides (TGs) and products from the lipolytic activity such as monoglycerides (MGs), diglycerides (DGs) and free fatty acids (FFAs) form part of the palm oil components [44]. TGs are mainly responsible for the physical characteristics of palm oil such as melting point, solid fat content and the induction time of crystallization [45]. Palm oil and palm kernel oil differ in their physical and chemical characteristics, although they come from the same fruit [45]. The major fatty acids content in palm oil are palmitic and oleic acids, while palm kernel oil contains mainly 46.0 to 51.0% lauric acid and is generally termed as lauric oil [46]. 

Palm kernel oil contains some nonglyceride components. These components are removed or reduced to acceptable levels in order to convert it to edible form. The nonglycerides are of two broad types: oil-soluble and oil-insoluble. The oil soluble nonglycerides such as free fatty acids, trace metals, phospholipids, carotenoids, tocopherols/tocotrienols, oxidation products and sterols are more difficult to remove from the oil, and require various refining steps [47]. 

## 5. Minor Constituents of Palm Oil/Palm Kernel Oil

Palm oil comprises of two categories of minor constituents. The first category of constituents is made up of derivatives of fatty acid including acylglycerides, mainly monoglycerides and diglycerides, phosphatides, esters, and sterols. The second category comprises of non fatty acid related compounds, specifically hydrocarbons, and these include aliphatic alcohols, free sterols, tocopherols and pigments. Crude palm oil serves as one of the richest sources of biologically active carotenoids and the largest natural source of tocotrienol, which is a part of the vitamin E family.

Since 1980s the presence of carotenes and vitamin E in palm oil has been well acknowledged [48]. These minor constituents of palm oil have drawn attention worldwide due to their industrial applications and beneficial health effects. Compared to other plants palm oil is the richest source of carotenoids and vitamin E content, ranging from 500 to 3,000 mg/kg each, depending on the species of palm fruit [49,50]. On the other hand, palm-pressed fiber oil contains high level squalene, phytosterols, carotenes and vitamin E, ranging from 1,102 to 4,638 mg/kg each, depending on whether fresh or dried fiber is used [51]. 

There are different types of carotenoids present in plants, bacteria, fungi and some animals. The various types of carotenes found in palm oil and fiber oil are summarized in Table 2 [52,53]. 

**Table 2:** Composition (%) of carotenes in palm oil and palm fiber oil [52,53].

Types of carotenes	Palm oil	Fiber oil
Phytoene	1.27	11.87
*Cis*-β-Carotene	0.68	
Phytofluene	0.06	0.40
β-Carotene	56.02	30.95
α-Carotene	35.16	19.45
*Cis*-α-Carotene	2.49	1.17
ζ-Carotene	0.69	7.56
γ-Carotene	0.33	2.70
δ-Carotene	0.83	6.94
Neurosporene	0.29	3.38
β-Zeacarotene	0.74	0.37
α-Zeacarotene	0.23	Trace
Lycopene	1.30	14.13

Plant fruits are the major sources of carotenoids, which are antioxidants and widely used as natural colorants in the food processing industry [54]. Most recently, Machmudah *et al.* reported that carotenoids have been used as medicines for the treatment of cancer, cardiovascular disease and as immune system regulators [55]. Furthermore, carotenoids have been shown to be beneficial to human health, especially in the role of pro-vitamin A which is known to prevent xerophthlamia, a hardening of eye tissue related to night blindness.

Among all other carotenoids, β-carotene is commonly and commercially used in food processing industry for its excellent coloring properties. β-carotene is a precursor of vitamin A and plays an important role in human health as well as the cellular regulatory system [56]. Apart from its colorant properties, β-carotene is known to have several other physiological functions, including antioxidant activity and inhibition of colon cancer cell growth [57]. It also has pharmaceutical, cosmetic and therapeutic uses [58]. Human beings cannot synthesis carotenoids in the body, so they need intake from food sources. 

Lycopene is another important carotenoid. Lycopene (C_40_H_56_) consists of a long chain hydrocarbon with conjugated 11 carbon-carbon double bonds. It is a natural red pigment and has extensive applications in the nutraceutical, pharmaceutical and in cosmetics market due to its natural deep-red pigments. Furthermore, it has potent antioxidant and anticarcinogen activity [59].

It is well known that palm oils as well as palm-pressed fiber oils are rich in vitamin E. Tocopherols and tocotrienols are the major vitamin E isomers found in crude palm oil as well as palm-pressed fiber oil. The average vitamin E concentration in fiber oil is 2,882 mg/kg [57]. Of all vitamin E isomers, tocotrienols constitute about 70–80% of total vitamin E present in crude palm oil [53]. However, in palm-pressed fiber oil, the major compound is α-tocopherol, which is about 60–70% [46]. Natural tocopherols and tocotrienols are well absorbed by body tissue, therefore extraction of these components from natural sources is of interest [60]. Moreover, there is growing interest in tocopherols and tocotrienols in the food industries due to their antioxidant properties and other nutraceutical effects [61]. Tocopherol and tocotrienol have been shown to possess antitumor activity and also to reduce cardiovascular disease (CVD) [62]. In addition, tocopherols are used commercially to fortify food or as nutritional supplements. Most of the natural vitamin E is used for human consumption, while only a small portion is used for animal feed and cosmetics [63]. 

Phytosterols are used as intermediates for the synthesis of hormones and drugs and widely used as cholesterol-lowering agents in food and pharmaceuticals industries. Lau *et al*. [57] reported the highest concentration of sterols 10,877 mg/kg and squalene 9,690 mg/kg in palm oil. β-sitosterol is the major constituent at 70% [51] and is potentially hypocholesterolemic [64]. Squalene acts as chemopreventive agent against some type of cancers [65,66].

## 6. Major Uses of Palm Oil and Palm Kernel Oil

Palm oil and palm olein are used mainly as frying oils in the food industry around the World. They are widely used in large-scale industrial frying for the preparation of doughnuts, instant noodles, crisps, and chips and commercial frying of snack foods. Palm oil is an ideal oil due to its composition, natural consistency, appearance, pleasant smell and heat resistant nature. It is comparatively cheap to use and produce fried food products with good flavor and long shelf life. Palm oil contains only a moderate amount of the more stable linoleic acid (10–12%). Palm olein is used both in the food industry and in homes and restaurants for frying and cooking. Palm olein produces less smoke, less foam and does not polymerize to gums compared to polyunsaturated oils. However, on repeated frying, a brown color is formed from the phenolic minor components in palm oil products but this color formation is unrelated to any deterioration of the fat. Because of the unique ratio of saturated to unsaturated TG, palm oil can be fractionated easily to a solid fat (stearin) and liquid oil (olein). These fractions can then be manipulated in different proportions to serve as raw materials for margarine production. Nowadays, margarines of different kinds are required to meet the varying demands by the food industries [67]. Hard margarine, soft table margarine and puff pastry margarine are some of the products made out of palm oil. There are many different kinds of palm-based shortenings, each tailor-made for a particular application. 

PKO are widely used for the manufacture of margarine, cocoa butter substitutes and other confectionery fats, biscuits or cookies with filling creams, cake frostings, imitation whipping cream and many other fascinating food products [68]. It is also used as replacement of butterfat in various dairy products such as ‘filled’ milk (e.g., coffee ‘whitener’ and coffee ‘creamer’), ice cream and cheese.

The Malaysian palm-based oleochemical industry has been advancing rapidly and is producing an increasing number of products like fatty acid methyl esters, fatty alcohols and glycerine [69]. In 2000, Malaysia produced 1.2 million tons of palm based oleochemicals, contributing to 19.7% of the total global production [70].

**Figure 1:**
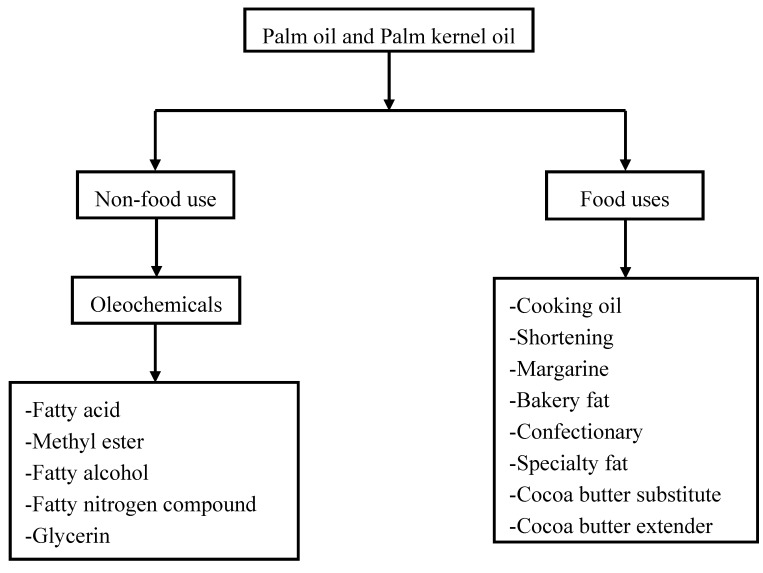
Flow diagram showing the usage of palm oil and palm kernel oil.

The fatty acid composition of palm kernel oil is very similar to that of coconut oil, while the fatty acid compositions of palm oil and palm stearin are similar to that of tallow [12]. Technically, therefore, palm kernel oil and palm oil could, to a large extent, replace coconut oil and tallow in the manufacture of oleochemicals provided availability and cost competitiveness are satisfactory. Oleochemicals are derived mainly from fatty acids and these can be manufactured from any of the oils and fats, but the ones actually used in the oleochemical industry are determined by market demand for particular types of fatty acids and their derivatives. All oils and fats can be converted to soap and the particular oil or fat used will determine the properties of the soap; and these properties of the soap are generally a function of the fatty acids. For the production of the best soaps no single oil or fat is suitable; a mixture of oils or fats has to be used. PKO closely resembles coconut oil and it is suitable in the production of high-quality soap. The major uses of palm oil and palm kernel oil are shown in Figure 1. 

## 7. Conventional Extraction of Palm Oil and Palm Kernel Oil

In Malaysia, three methods are being used for extracting palm oil and palm kernel oil: screw press, direct solvent extraction, and pre-pressing followed by solvent extraction [46]. The screw press technology utilizes large quantities of water to sterilize the palm fruits as a pretreatment process. The process wastewater is then discharged as palm mill oil effluent (POME) and is harmful to the environment [71]. The solvent extraction processes can be divided into three main unit operations; kernel pre-treatment, oil extraction and solvent recovery from oil and meal. In this process the pre-treated kernel flakes are first pre-pressed, leaving about 15 to 20% oil in the pre-expelled cake. Finally the cake is solvent extracted to remove the remaining oil. 

Physical and chemical treatments are two types of processes used to refine crude palm oil. Generally, physical refining is the more popular and advantageous method because it is the most economical means for removing undesirable substances and avoid concomitant environmental concerns. Typically, physical refining consists of three major steps. Degumming is the first step to remove undesired gummy materials such as phosphatides. Secondly, the bleaching step is involved to remove color pigments and the final step is deodorization process to get rid of the unpleasant odor and taste due to the presence of aldehydes and ketones. Deodorization of FFAs is by steam distillation at 270 °C under vacuum and the final product will be refined, bleached, and deodorized oil which requires three separate mills in addition to the logistics and cost in the processing. In other cases, organic solvents like hexane have been used to extract the oil. Moreover, some valuable nutrients such as tocopherols and carotenes present in palm oil are also destroyed during the removal of FFA via deodorization [8]. List *et al.* [7], reported that about 99.2% of the gums removed from crude soybean oil by SC-CO_2_ degumming process while conventional water degumming removes 80–95%. They also reported that after SC-CO_2_ degumming crude soybean oil flavor stability is better than that of commercial refined oil. Manan *et al.* [9], described the modeling of a new SFE process for palm oil refining that can overcome the limitations of the existing refining processes. In comparing direct expelling, direct solvent extraction and pre-expelling/solvent extraction different opinions have been expressed regarding the most economical palm oil extraction method. Some feel direct solvent extraction is suitable for oilseeds containing less than 20% oil, while per-pressing followed by solvent extraction is used for high oil content seeds (exceeding 20%).

## 8. Applications of SFE in Palm Oil and Palm Kernel Oil Extraction

### 8.1. Supercritical Carbon Dioxide (SC-CO_2_) Extraction of Palm Kernel Oil

Many researchers have investigated the extraction and fractionation of palm kernel oil from palm kernels using SFE [43,72,73,74,75,76]. The yield of PKO was shown to increase with pressure (34.5 to 48.3 MPa at 353.2 K). At lower pressure (20.7 to 27.6 MPa), lower amounts of shorter chain TGs (C8-C14) were obtained. More longer chain FA constituents (C16-C18:2) were extracted at higher pressures from 34.5 to 48.3 MPa. At higher temperature, the SC-CO_2_ extracted PKO was found to be superior in terms of constituent fatty acids in TG compared to that obtained from Soxhlet extraction using hexane. Table 3 shows the TGs composition in PKO extracted by Soxhlet using hexane and by SC-CO_2_ extraction at different temperature and pressure [43].

**Table 3:** Fatty acid constituents in palm kernel oil (PKO) extracted using Soxhlet and SC-CO_2_ at different pressure and temperature 313.2 K and 353.2 K. Reproduced with substantial modification from [43].

Temperature (313.2 K)									Temperature (353.2K)								
Pressure (MPa)	Fatty acids constituents (%)								Pressure (MPa)	Fatty acids constituents (%)							
	**C**_8_	**C**_10_	**C**_12_	**C**_14_	**C**_16_	**C**_18:0_	**C**_18:1_	**C**_18:2_		**C**_8_	**C**_10_	**C**_12_	**C**_14_	**C**_16_	**C**_18:0_	**C**_18:1_	**C**_18:2_
20.7	6.9	6.0	52.6	16.5	8.5	0.5	8.9	0.1	20.7	7.0	6.0	52.8	16.6	8.8	0.3	8.4	0.1
27.6	6.2	5.4	52.1	16.0	8.1	0.9	10.5	0.8	27.6	6.4	5.6	52.9	16.3	7.3	0.8	10.1	0.7
34.5	5.9	5.3	51.1	15.5	8.9	1.0	11.8	0.8	34.5	5.0	4.9	50.0	14.4	10.0	1.4	12.9	1.4
41.4	5.3	5.0	51.6	14.6	9.8	1.1	11.9	0.9	41.4	4.1	4.0	48.0	12.7	11.9	2.1	14.5	2.8
48.3	4.6	4.0	48.2	13.1	11.2	1.9	13.7	3.4	48.3	3.3	3.1	42.9	9.1	14.9	2.5	19.0	5.1
Soxhlet extraction	4.0	3.7	48.0	15.4	7.5	2.0	15.1	2.7	Soxhlet extraction	4.0	3.7	48.0	15.4	7.5	2.0	15.1	2.7

A pressurization-depressurization SC-CO_2_ extraction technique (pressure swing technique) was used to separate PKO from undehulled ground palm kernel [38]. The PKO yields obtained by using combined pressure swing (PS) and continuous extraction are shown in Table 4. The authors compared these results with those of continuous SC-CO_2_ extraction and combined PS extraction. Pressure swing extraction gave higher yield compared to continuous extraction at all pressures (Table 4). Moreover, they reported that the yield was doubled when PKO was extracted using a combined PS process for any given amount of CO_2_ used. They also claimed that, almost all of the oil from palm kernel particles extracted with combined pressure swing and continuous extraction with the use of a low amount of CO_2_. The highest total oil yield 46.9% was reported for the combined PS technique at 25 MPa with CO_2_ flow rate of 2.49 g min^−1^. In comparison, the yield of the continuous extraction was lower (34.9%) than the combined PS extraction at same pressure and carbon dioxide used. 

**Table 4:** Yield of both combined pressure swing (PS) extraction and continuous extractions at various time intervals up to total extraction times of 150 min. Reproduced with permission from Elsevier [38].

Step	Time (min)	Yields (%)							
		Pressure swing extractions, pressure (MPa)				Continuous extractions, pressure (MPa)			
		10	15	20	25	10	15	20	25
1	10	1.6	2.8	4.5	9.9	0.8	1.2	2.4	3.9
2	10	1.7	3.8	5.0	12.0	0.9	1.2	2.5	3.9
3	10	1.7	2.7	3.3	4.6	1.0	1.4	2.4	4.1
4	120	13.8	19.8	24.2	20.4	8.2	12.9	19.5	23.0
Total	150	18.8	29.1	37.0	46.9	10.9	16.7	26.8	34.9

Note that step 4 consists of 120 min of continuous extraction for both PS extraction and continuous extraction.

The color and fatty acid composition of the extracted PKO also varied with extraction pressure and temperature [76]. The variation in oil composition and thereby the characteristics such as slip melting point and solid fat content qualified each fraction to be used in different products, such as in margarine formulations and as a cocoa butter substitute. At a higher pressure and temperature of 48.3 MPa and 80 °C respectively, the color and composition of the fractions did not change significantly, indicating low selectivity of SC-CO_2_, whereas, the extraction rate and solubility of PKO in SC-CO_2_ increased. Thus, a better and efficient extraction and fractionation process can be expected if the system is operated at higher pressures using multiple pressure reduction collectors [76].

Zaidul *et al.* [18] found some variations in the percent of fatty acids in different fractions of palm kernel oil. Shorter chain fatty acids (caprylic, capric, lauric and myristic acids) were found to decrease from the first to the last fraction but long chain fatty acids (palmitic, stearic, oleic and linoleic acids) increased from the first to the last fraction as the extraction period extended [18]. Thus, SC-CO_2_ can obviously be applied in the selective reduction of short chain fatty acids in a particular fraction of extracted PKO. Similar results were reported for PKO and palm oil fractions by Markom *et al.* [77] and Norulaini *et al*. [75]. Norulaini *et al.* [74,75], studied further reduction of lauric acid and enhancement of oleic and stearic acids in PKO and was able to produce low-lauric or non-lauric confectionery fat.

### 8.2. Supercritical Carbon Dioxide (SC-CO_2_) Extraction of Residual Palm Kernel Oil from Palm Kernel Cake to Produce Palm Kernel Fiber

In palm oil mills, the residues remaining after the oil extraction is known as palm-pressed fiber and palm kernels. The application of SFE to palm-pressed fiber could be a novel process to recover the residual oil in the palm pressed fiber. The resulting defatted palm pressed fiber can be a good source of edible fiber for human consumption. Moreover, the residual palm kernel meal, a by-product obtained from the SC-CO_2_ extraction process, is suitable for use as poultry feed owing to the reduced fiber content of the kernels [18].

The palm-pressed fiber is sterilized in order to inactivate bacteria and deactivate the lipases to prevent the enzymatic hydrolysis of lipids which releases FFAs [51]. Norulaini *et al.* [71], found that bacteria were completely removed when the fiber treatment were conducted at higher than the standard sterilization conditions. Several groups of researchers carried out the extraction of palm oil from palm press ﬁber [51,71]. The quality aspects, minor components and acylglycerols of fresh and dried palm-pressed fiber oil extracted using SC-CO_2_ and hexane are given in Table 5 [51]. The quality of oil recovered from fresh palm-pressed fiber was generally better than the oil recovered from dried fiber. The concentrations of minor components in dried fiber oil were higher compared to fresh fiber oil for all extraction methods. The α-tocopherol content was higher in dried fiber while γ-tocopherol was found to be higher in fresh fiber.

Palm-pressed fiber oil contains palmitic acid as the major FA, followed by oleic acid. Palmitic acid showed higher yield at lower pressure (13.7 MPa), but oleic acid reflected higher yield at higher pressure (27.6 MPa). Linoleic acid required the highest pressure (34.5 MPa) as tested in Lau *et al.*’s study [51]. The variation in FA composition of SC-CO_2_ extracts is due to variation in solubility of different fatty acids at variable extraction conditions. Higher pressure is needed to dissolve the longer chain FAs (C18:0, C18:1, C18:3, C20:0 and C20:1) relative to short and medium chain FAs (C8, C10, C12 and C14).

**Table 5:** The physico-chemical characteristics, minor components and acylglycerol contents in the SC-CO_2_ and hexane extracted palm fiber oil ^a^. Reproduced with substantial modification from [51].

Sample	Fresh fiber			Dried fiber		
Extraction method	SC-CO_2_	SC-CO_2_	hexane	SC-CO_2_	SC-CO_2_	hexane
	(50 °C, 30 MPa)	(80 °C, 30 MPa)		(50 °C, 30 MPa)	(80 °C, 30 MPa)	
Physico-chemical characteristics						
DOBI (%)	2.21 ± 0.12	2.19 ± 0.09	2.01 ± 0.08	2.69 ± 0.05	2.58 ± 0.10	2.08 ± 0.07
OSI (h)	21.9 ± 0.15	18.8 ± 0.11	>48	16.6 ± 0.32	16.1 ± 0.22	33.7 ± 0.34
PV (meq O_2_/kg)	0.46 ± 0.03	0.52 ± 0.03	0.84 ± 0.04	1.83 ± 0.05	2.34 ± 0.06	2.23 ± 0.04
FFA (%)	3.46 ± 0.05	3.84 ± 0.04	3.94 ± 0.03	3.79 ± 0.05	3.78 ± 0.06	3.94 ± 0.03
Minor component (mg/kg)						
Carotenes	2909 ± 15	3424 ± 13	2933 ± 11	4424 ± 16	4638 ± 21	4007 ± 15
Vitamin E	1979 ± 14	2372 ± 14	1981 ± 18	2303 ± 19	2584 ± 17	2251 ± 13
Phytosterols	4429 ± 31	4409 ± 27	4349 ± 33	4749 ± 31	4568 ± 19	4610 ± 22
Squalene	1102 ± 21	1321 ± 18	1117 ± 20	1642 ± 22	1633 ± 12	1495 ± 25
Acylglycerol content (%)						
MAG	0.35 ± 0.02	0.38 ± 0.03	0.37 ± 0.04	0.31 ± 0.02	0.28 ± 0.03	0.27 ± 0.02
DAG	0.85 ± 0.02	0.89 ± 0.05	1.14 ± 0.03	0.86 ± 0.04	0.83 ± 0.04	0.92 ± 0.02
TAG	94.30 ± 1.03	93.73 ± 0.79	93.52 ± 1.12	93.73 ± 0.32	93.78 ± 0.77	93.87 ± 0.62

Each sample was analyzed in triplicates ^a^. DOBI: Deterioration of bleachability index, OSI: Oxidative stability index, PV: Peroxide value, FFA: Free fatty acid, MAG: Monoacylglycerol, DAG: Diacylglycerols, TAG: Triacylglycerols.

### 8.3. Supercritical Carbon Dioxide (SC-CO_2_) Fractionation of Palm Kernel Oil

Selective extraction of low vapor pressure oils can be done using supercritical fluids which is not easily attainable by distillation. These oils cannot be fractionated by distilling because of the presence of impurities with equal volatility as the main components, thus failing to achieve good fractionation. Fractionation using supercritical fluid with respect to chemical composition to produce oil fractions with different carbon lengths and saturations has been investigated. Hassan *et al.* [76], fractionated PKO through supercritical carbon dioxide extraction. For the extraction and fractionation of PKO, the SC-CO_2_ was used as a solvent in the pressure range 20.7–48.3 MPa and temperatures between 40 and 80 °C. At lower pressures 20.7 and 27.6 MPa, the solubility of PKO in SC-CO_2_ decreased with temperature while at higher pressures of 34.5, 41.4 and 48.3 MPa, the solubility increased with temperature. The authors found that the earlier fractions rich in short-chain triglicerides, while the later fractions were rich in longer chain triglicerides and unsaturated triglicerides. The authors also reported that the short chain fatty acid contained in PKO are easily soluble in SC-CO_2_.

### 8.4. Supercritical Carbon Dioxide (SC-CO_2_) Palm Kernel Oil Fractions to Make Cocoa Butter Replacers (CBRs)

Supercritical carbon dioxide is a convenient solvent for the fractionation of PKO, reducing shorter and medium chain (C_8_-C_14_), and increasing the longer chain (C_18:0_-C_18:2_) fatty acid constituents in PKO [72,75]. Many researchers have fractionated fatty acid triglycerides based on carbon number using SC-CO_2_ in the temperature range of 40–80 °C and pressures up to 50 MPa [72,74,75,76,78,79]. Zaidul *et al.* [68], studied the blending of fractionated PKO obtained by SC-CO_2_ extraction and palm oil for the production of CBRs. In that study a total of 10 blends were studied. The authors have successfully produced CBRs in respect to the physio-chemical properties such as fatty acid constituent, slip melting point (SMP), iodine value (Iv), saponification value (Sv), acid value (Av), cloud point (Cp) and solid fat content (SFC).

### 8.5. Supercritical Carbon Dioxide (SC-CO_2_) Extraction of Oil from Solid Matrix

SCF or near SCF extractions of palm oil fruits using palm oil liquid can be conducted at elevated temperatures. The underlying principal behind this process is the phase equilibrium of palm oil and supercritical palm oil as solvents to extract oil from oil palm fruits at various temperatures and pressures. A study beyond the critical points can be conducted in the near future. In this novel extractions of the palm oil as the soluble constituent, the extract must first be released from its bound state, thereafter diffuse through the porous structure and finally through the fluid layer. At present there is a lack of knowledge about the phase equilibrium for this system, solubility, diffusivity and the mass transfer rate for the overall process. In general, such rates are controlled by the combination of internal and external diffusion resistances. In order to effectively emulate the extraction processes, the solubility of the palm oil as the solute in the supercritical palm oil which acts as the solvent is the most important thermo physical property that must be determined. In particular, the pressure and temperature, as well as the density dependence of solubility must be determined. This information is necessary before the operating conditions can be determined.

In the palm oil industry, the objectives that are of significance to decision makers are to maximize crude palm oil and palm kernel oil production and to minimize losses of palm oil and palm kernel during processing and also to bring production costs to the lowest possible level. SCF technology has a number of advantages over the conventional processing methods for the extraction, purification and fractionation of palm oil [8,72]. These include low-temperature operation, selective separation, inert solvent, little wastewater and the extraction of a high-value product or a new product with improved functional or nutritional properties. Considerable attention has been given to the development of SCF processing in the oil and fat industry. However, use of SC-CO_2_ for the extraction of palm oil from its fruits has been found to be relatively rare as compared to SC-CO_2_ extraction of other vegetable oils.

### 8.6. Supercritical Carbon Dioxide (SC-CO_2_) Extraction of Oil from Mesocarp

There are few available palm oil studies reporting on the extraction [80] and fractionation [77] of the oil from the ﬂeshy mesocarp. Lau *et al.* found that the oil recovery from dried mesocarp was slightly higher (77.3%) using SC-CO_2_ compared to mechanical screw-pressing (74.2%) [80]. The free fatty acid content in SC-CO_2_-extracted palm oil was low (0.61%) when compared to conventionally extracted crude palm oil (3.15%). The quality of commercial crude palm oil (CPO) was determined by the measurement of bleachability index (DOBI), oxidative stability index (OSI), peroxide value (PV) and free fatty acid (FFA) value [80]. The determination of the quality of commercial CPO and CPO extracted using SC-CO_2_ and hexane is shown in Table 6 [80]. 

**Table 6:** The physico-chemical characteristics, trace metals, minor components and acylglycerol contents in the SC-CO_2_, hexane and commercial extracted palm oil. Reproduced with substantial modification from [80].

	Extraction method		
	SC-CO_2_	Hexane	Commercial CPO
Physico-chemical characteristics			
DOBI (%)	2.60	2.93 ± 0.24	2.75 ± 0.31
Oxidative stability index (OSI) (h)	14.79	16.08 ± 1.03	15.50 ± 0.96
Peroxide value (PV) (meq O_2_/kg)	1.68	1.47 ± 0.32	1.94 ± 0.27
Free fatty acid (FFA) (%)	0.612	0.371 ± 0.093	3.15 ± 0.35
Trace metal content (mg/kg)			
Iron	0.51	6.26 ± 1.02	6.06 ± 2.12
Copper	0.05	0.22 ± 0.05	0.12 ± 0.10
Minor component (mg/kg)			
Carotenes	972	906 ± 64	879 ± 72
Vitamin E	512	557 ± 42	614 ± 34
Phytosterols	826	768 ± 38	674 ± 21
Squalene	632	627 ± 39	524 ± 22
Acylglycerol content (%)			
MAG	0.14	0.15 ± 0.03	0.14 ± 0.04
DAG	4.16	4.32 ± 1.17	5.51 ± 1.62
TAG	94.95	94.81 ± 2.53	91.93 ± 2.17

The OSI of SC-CO_2_-extracted palm oil also was slightly lower than commercial and hexane-extracted crude palm oil because the pro-oxidants iron and copper (trace metals) were reduced in the SC-CO_2_-extracted oil but the overall quality of SC-CO_2_-extracted CPO appeared equivalent to that obtained by commercial processing of CPO (Table 6). In the palm oil industry, the extraction, fractionation and refining of commercial crude palm oil could be contaminated with iron and copper by the soil and wear and tear of the processing machinery [80]. On the other hand, the single processing operation of SFE is combined and very simplified for the extraction, fractionation and refining of palm oil, gives less opportunity to oil contamination or reduce the level of iron and copper in the oil. It can be concluded that the SFE technique may widely used in palm oil industries for trace metals-free good quality oil production. It was also found that triglycerides and minor components such as carotenes, phytosterols, and squalene in the SC-CO_2_-extracted palm oil are higher compared to hexane-extracted and commercially extracted palm oil. So, the study showed that the palm oil extraction by using SC-CO_2_ can potentially replace the conventional screw press extraction, clarification and vacuum drying processes [80].

SFE is able to fractionate, refine, and bleach. Deodorization from SFE extracted oil is done under steam after extraction. Using countercurrent SFE, the free fatty acid removal from crude oil can be done within a short period without any loss of triglyceride [81]. Manan *et al.* successfully designed a new process for the production of refined palm oil based on SFE technology by using the Aspen Plus simulator [9]. This simulator may be used for stead state or transient calculations. This process was also found to be suitable for enriching tocopherols and to remove the FFA from CPO using SC-CO_2_ as solvent. 

Besides extraction and refining, simultaneous fractionation of palm oil by supercritical fluid is also possible. During extraction the initial fraction is solid and the latter fractions are semi-liquid and liquid at room temperature [77]. The solid appearance of the initial fractions is due to the high content of saturated fatty acid (mainly C16:0) rich triglycerides; they appear ﬁrst due to their high solubility in SC-CO_2_. The liquid fractions contain mostly triglycerides rich in unsaturated FAs (mainly C18:1). The concentration of the short chain and saturated FAs in palm oil decreases as the extraction time increases. Conversely, the concentration of the heavier and mainly unsaturated FAs increases with the progress in extraction, after most of the shorter chain FAs have been removed making these longer chains accessible to the SC-CO_2_.

## 9. SFE of Minor Constituent of Palm Oil

Minor components in palm oil such as carotenoids, vitamin E, sterols, squalene and others are mostly removed from the oil during traditional oil refining procedures [82]. Palm oil has been used to extract these natural valuable compounds by employing various methods. They include urea processing, saponification, separation by adsorption, crystallization, selective solvent extraction, molecular distillation and transesterification followed by molecular distillation [49,83]. However, the difficulty and inefficiency of most of these methods are ascribed to the sensitivity of carotene to light, oxygen, heat and acid degradation, low content in all natural sources, and low selectivity of separation techniques due to the similar physical properties (polarity, solubility, molecular weight) of the various carotenes [58]. Moreover, use of organic solvent in these methods which are potentially harmful and cost effective. SC-CO_2_ extraction has become an increasingly popular method for the valuable natural minor components of palm oil, due to its distinctive advantages, as well as low temperature use, selective extraction, simpler and cleaner (solvent-free) product recovery as well. The application of supercritical fluid extraction of carotenes and vitamin E from palm oil is seen to have great potential for replacing the conventional screw-press extraction, clarification and vacuum drying processes [51]. Several other reports are available on the SC-CO_2_ extraction of carotenoids and vitamin E from palm and palm-pressed fibers oil [8,71,84]. Furthermore, The SC-CO_2_ extraction of carotene and the impact of pressure, temperature and time have been extensively studied by many researchers [52,57,85,86,87,88].

Lau *et al.* [57], extracted two fractions of fiber oil enriched with vitamin E and carotene, respectively from fresh palm-pressed mesocarp fiber using SC-CO_2_ at 40 °C in three steps by a continuous extraction technique. About 40% of the triglyceride with low carotene content was obtained at 10 MPa and 20 MPa in the earlier fraction, retaining maximum carotene that enriched the latter fractions with average concentration of 3,942 mg/kg and 90%. The vitamin E was extracted with highest concentration of 3,650 mg/kg in the first fraction, while carotene was enriched to 5,498 mg/kg in the later fraction. From their study, it has been shown that the solubility of vitamin E in SC-CO_2_ is higher than that of carotene. Therefore, carotenes can be separated from other minor components based on solubility differences by manipulating the SC-CO_2_ extraction parameters.

To date, the solubility of carotenoids and vitamin E in supercritical carbon dioxide and the effect of pressure, temperature have been widely studied. Wei *et al.* [52], reported the effect of pressure, temperature, on the solubility of palm carotenoids. They found that the solubility of carotenoids in SC-CO_2_ is affected by increasing pressure at the constant temperature or decreasing temperature at constant pressure. However, increasing flow rate and decreasing sample size can reduce extraction time. The solubility of carotenoids found in their study is in the range between 1.31 × 10^−4^ to 1.58 × 10^−3^ g kg^−1^ at different pressure range 14 to 30 MPa while lower solubility was also reported by Johannsen and Brunner with 1 × 10^−3^ to 4 × 10^−2^ g kg^−1^ [88].

Tocopherol and carotene differ greatly in their solubility in SC-CO_2_. The solubility of squalene and sterols in supercritical fluids was studied by Lau *et al.* [57]. In their study, the solubility of squalene in the mixture at 10 MPa was 0.189 mg/g CO_2_ while that of sterols was 0.132 mg/g CO_2_, even though the initial concentration of sterols (4,349 mg/kg) was four times higher than that of squalene (1,117 mg/kg). Squalene showed high solubility in low density SC-CO_2_ (10 MPa) with a 77% recovery during the 1st 4 h of extraction. In that study, squalene showed the highest solubility among all other components due to its nonpolar characteristic and smaller molecular size. Thus, the SC-CO_2_ technique has the potential to produce two types of value-added oils from palm-pressed mesocarp fiber. 

## 10. Prospect and Limitation of SC-CO_2_ in Palm Oil Industry

Laboratory scale attempts are always the primary steps to establish a new technique or methodology. As an attempt to industrialization of SFE technology, SCF has been applied to a lot of extraction purposes at the laboratory scale and its suitability has been reported. Casas *et al.* [89], reported that SFE can be applied from a laboratory scale to large industrial scale (less than a gram to tons of raw materials). Before using in the industry, a method or technology needs scaling-up from laboratory to pilot plants. Successful scale up means the maintenance of quality of products obtained after scale up. Typically before SFE is employed at the industrial scale, a pilot plant is set up to test the soundness of the system incorporating laboratory data in the design. The easy scale-up procedure for SFE processes consists of two steps: the first is to perform small scale assays to define the optimal extraction conditions through a screening of operational parameters. The second step involves the selection of the scale-up method based on the kinetic limiting factors [90]. Reverchon *et al.* [91], compared lab-scales and pilot scales in supercritical assisted atomization. In most cases the parameters to be evaluated are extraction pressure, CO_2_ flow rate and particle size. There are several mass transfer models to explain the extraction curves among which logistic model, diffusion model and Sovová model are the most convenient [92,93]. Furthermore, Oliveira *et al*. [94] reviewed significant models such as the linear driving force, shrinking core, broken and intact cells (BIC), and the combination of BIC and shrinking core models. 

Recently, the kinetics and mathematical model described by Mezzomo *et al.* [90], for scaling-up of the SFE method was used for extraction of peach almond oil. This SFE modeling defined some useful parameters for process design, especially in equipment dimensions, solvent flow rate and particle size determination. Casas *et al.* [89], successfully established scaling-up of the SC-CO_2_ method for the extraction of bioactive compounds from the sunflower leaves, where the authors investigated the influence of the extraction time at various flow rates of SC-CO_2_ solvent. Aro *et al.* [95], reported a study using pilot scale SFE equipped with two extraction chambers, where one chamber was for antisolvent process for the production of pure phospholipids from egg yolk. Pettinello *et al.* [96], scaled up a supercritical fluid chromatography for purification of eicosapentaenoic acid ethyl ester (EPA-EE) where the pilot scale purity of EPA-EE reduced from 95% (bench scale) to 93%.

**Figure 2:**
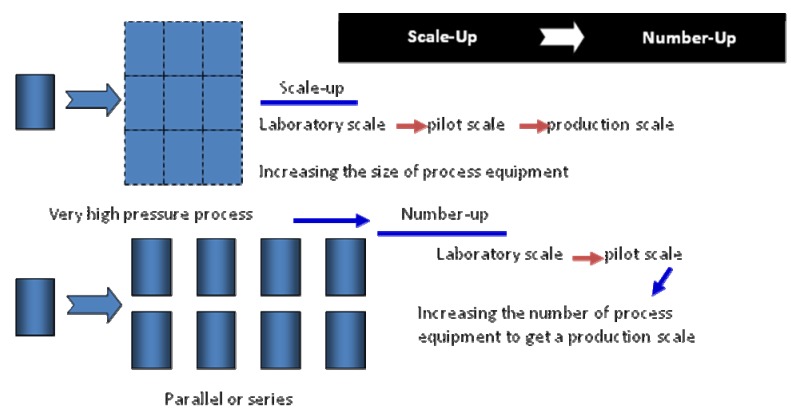
Scale-up to larger, geometric full-scale system.

In the near future studies number-up could be used over the scaling-up method using geometric, dynamic, and kinematic similarities. This method is introduced by Oldshue in 1983 [97]. Figure 2 shows the overall concept of Number-up method.

Palm oil is the oil with the lowest production costs, considering that palm oil is now the “marginal” oil [98]. Further decrease in production cost by using SFE will push palm oil one step forward in taking over the world market. The move away from *trans*-fatty acids also favors palm oil, because it can be used without hydrogenation as the solid fat component in many formulations [99]. 

As discussed in earlier sections, SFE could be advantageous for fractionating different constituents of palm oil and PKO and be blended again in desired ratios to formulate different types of palm oil according to the market demands. SC-CO_2_, being a cheaper recyclable solvent, will ensure it will not be costly to produce the desired fractions and bears no threat to health and the environment. The major hurdle to the application of SFE or SC-CO_2_ in palm oil industry is its instrumentation on an industrial scale. Another problem is the maintenance of the SFE equipment which is somewhat costly as gas-leakage can be frequent during high pressure operation. Attention should be paid while constructing the heavy duty equipment for use in the industry. It is also important to keep in mind during construction of the equipment to link a supercritical fluid chromatography to SFE equipment to be used for special purposes to concentrate desired components. By this time SFE has been established to industrial scale in essential oil extraction from different spices and in extraction of some pharmaceutical or phytochemicals from different plant parts. Once the industry adopts SFE equipment in palm oil extraction, the process can appear cost-effective or more profitable than the conventional methods.

## 11. Supercritical Fluid Extractions of Other Oil Types from Various Sources

Recently, Temelli *et al.* [25], reported and classified specialty oils such as nut oils (almond, hazelnut, peanut, pecan, pistachio, and walnut), seed oils (apricot, borage, cherry, evening primrose, flax, grape, hiprose, pumpkin, sea buckthorn, sesame, *etc*.), cereal oils (amaranth, oat, rice bran, and wheat germ), and fruits and vegetables oils (buriti fruit, carrot, cloudberry, olive husk, and tomato), which have been extracted using SC-CO_2_. The major advantage of the SC-CO_2_ extraction method for specialty oils is preserving the unique flavour and aroma while volatile aroma compounds are often lost during traditional solvent extraction processing. Specialty oils contain high level of bioactive components, such as polyunsaturated fatty acids, tocopherols, tocotrienols, phytosterols, carotenoids and squalene. These components play tremendously positive roles in human health. Due to the public awareness as well as demand food industries are always looking for such kind of oils which are naturally extracted. Meanwhile, SC-CO_2_ extraction has been demonstrated the best method for bioactive lipid components in the literature [100,101,102,103].

Essential fatty acids such as linoleic acid are necessary for human metabolism and cannot the synthesized inside the human body. Essential fatty acids must be supplied externally from the diet. Essential fatty acids play an important role in the formation of cell membranes, and the proper development and functioning of the brain and nervous system. Hormone-like substances called eicosanoids produced by essential fatty acid are responsible for regulating blood pressure and viscosity, vasoconstriction and immune and inflammatory responses [104]. It has been well demonstrated that fatty acid deficiency is associated with several human diseases. Fatty acid consumption has large health benefits such as reduced inflammatory diseases [105]. SC-CO_2_ has been extensively used to obtain oil as well as fatty acid components from various seeds: apricot [106], palm kernel [43], canola [107], rapeseed, soybean, and sunflower [108], jojoba [109], sesame [110], parsley [111], amaranth [112], borage [113], flax [114], and grape [115]. Moreover, oils have also been extracted from several nuts such as almond [116], walnut [100], peanuts [117], pistachio [118] and acorn [119]. Recently, several researchers have investigated SC-CO_2_ extraction of plants oil such as *Ferulago angulata* [120], *Sacha inchi* [121], chia [16], *Anastatica hierochuntica* [122]. SC-CO_2_ extraction of oils and the impact of various extraction parameters were also reported by many reporters in the literature. Supercritical fluid extraction of oils from various sources in different studies is shown in Table 7.

**Table 7:** Summary ofoil extraction from various natural sources using SC-CO_2_.

Samples	Scientific name	Extract	Oil yield (wt. %)	Pressure/Temperature (MPa)/T (°C)	References
**Seeds**					
Palm Kernel	*Elaeis guineensis*	Fatty acid composition	49	20.7–48.3/40–80	[43]
Peach seed	*Prunus persica*	Oils, fatty acid composition, tocopherols	70	15.0–19.8/40–51	[104]
Apricot	*Prunus armeniaca* L.	Oils		30–60/40–70	[106]
Canola seed	*Brassica napus*	Seed oil	19.49	20–25/40–60	[107]
Rapeseeds	*Brassica*	Seed oil	39.3	32–35/17–40	[108]
	*napus* var. Rapora				
Soybean	*Glycine max* var. Corsoy	Seed oil	16.6	28–30/2–40	[108]
Sunflower	*Helianthus annuus* var.	Seed oil	36	25–35/20–50	[108]
	Fransol				
Jojoba seed	*Simmondsia chinensis*	Seed oil	80	25–45/67–90	[109]
Sesame seed	*Sesamun indicum* L.	Seed oil	35	19–25/40–60	[110]
Parsley	*Petroselinum sativum* Hoffm.	Seed oil		10–15/35–45	[111]
Amaranth seed	*Amaranth cruentus*	lipids	7.95	10–30/35–50	[112]
Borage seed	*B. officinalis* L.	Seed oil	29	5–35/10–60	[113]
Flaxseed	*Linum usitatissimum* L.	Seed oil	35.3	30/50	[114]
Grape seed	*Vitis vinifera*	Fatty acid composition	13.6		[115]
Cardamom	*Elettaria cardamomum* Maton	Fatty acids, tocopherols Carotenoids, Chlorophylls, volatile constituents	6.65	10–80/25–35	[116]
Cottonseed	*Gossypium sp.*	Oil	17.26	3–55/60–80	[124]
Kenaf seeds	*Hibiscus cannabinus L.*	Seed oil	20.18	20–60/40–80	[125]
Passiflora seed	*P. edulis Sims var. edulis*	Seed oil	25.83	17–33/47–63	[126]
Pomegranate seed	*Punica granatum L.*	Fatty acid composition	3.39	20–35/40–60	[127]
Pumpkin	*Cucurbita maxima*	Seed oil	30.7	15–35/35–75	[128]
Rosehip seed	*Rosa canina* *L.*	Seed oil	15.93	15–45/40–80	[129]
Sea buckthorn	*Hippophaë thamnoides* L.	Oil, Vitamine E, Carotenoids		20–40/40–60	[130]
Chia seed	*Salvia hispanica L.*	Seed oil	92.8	25–45/40–80	[16]
Sacha inchi seed	*Plukenetia volubilis L.*	Seed oil, Tocopherols,	50.1	30–40/40–60	[121]
		Carotene			
Pithecellobium jiringan seed		Fatty acid, vitamin E, flavonoids		60/80	[131]
Aniseed	*Pimpinella anisum* L.	Essential oil	10.67%	8–18/30	[132]
Coriander seed	*Coriandrum satium* L.	Essential oil		20–30/35	[133]
Cherry seeds	*Prunus avium* L.	Essential oil		18–22/39–60	[134]
Evening primrose	*Oenothera biennis* L.	Essential oil		20–70/40–60	[135]
Guava	*Psidium guajava* L.	Seed oil	17.30	10–30/40	[136]
Orange peel		Essential oil		8–28/20v50	[137]
**Nuts**					
Walnut	*Juglans regia* L. *var.* Franquette	Fatty acid composition	19	20–40/50–70	[100]
Almond		Almon oil	50	35/40	[116]
Pistachio	*Pistacia Vera* L.	Essential oil	66	20.7–34.5/50–70	[118]
Acorn	*Quercus rotundifolia* L.	Fatty acid composition		12–21/35–60	[119]
Coconut	*Cocos nucifera* L.	Fatty acid composition		20.7–34.5/40–80	[138]
Hazelnut		Oil	33	15–60/40–60	[139]
**Cereal oils**					
Wheat germ		Fatty acid composition	10.15	20–35/40–60	[140]
Rice brain		Fatty acid composition	22	17–31/1–60	[141]
Oat		Digalactosyldiacylglycerols		40/50–70	[142]
**Plants**					
*Anastatica hierochuntica*	*Anastatica hierochuntica*	hexadecanoic acid, 9,12-octadecadienoic acid, heneicosane, heptacosane	1.15	22–46/32–46	[122]
Ferulago Angulata		Essential oil	1.0	9–19/35–55	[120]
Vetiver roots	*Vetiveria zizanioides* L.	Essential oil	5.90	10–19/40–50	[143]
*Nitraria tangutorum*		Seed Oil		10–30/30–50	[144]
yellow horn	*Xanthoceras sorbifolia* Bunge	Seed oil	61.28	15–35/30–60	[145]
Rose geranium	*Pelargonium* sp.	Essential oil	77.82	8–16/40–100	[146]
Silkworm pupae		Essential oil	29.73	15–35/30–50	[147]
Sage leaves	*Salvia Officinalis* L.	Essential oil	1.35	8–10/45–60	[2]
Lovage	*Levisticum officinale* Koch.	Essential oil	4.92	8–35/40–50	[148]
Chamomile	*Chamomilla recutita* [L.] Rauschert	Essential oil	4.33	10–20/30–40	[149]
Lemongrass	*Cymbopogon citratus*	Essential oil	1–2%	8.5–12/23–50	[150]
Black pepper	*Piper nigrun L.*	Essential oil	70	15–30/30–50	[151]

## 12. Conclusions

Supercritical fluid technology has made significant advances for the extraction of oil from natural sources over the past 20 years. The palm oil industry is a prospective field for the application of SFE method using SC-CO_2_ as a solvent that can minimize wastewater compared to conventional mechanical extraction. It is a cost effective technique at both the laboratory- and industrial-scale for the extraction of palm oil, palm kernel oil and minor components such as carotenes, tocopherols, and more from palm oil and palm leaves. Extraction of tocopherols from leaves taken off the palm trees while collecting the fruits may be a route to be evaluated further. In general, tocopherol levels in palm leaves were not high enough to allow an economic industrial-scale extraction. SFE can also act as a sterilizer for palm fiber oil, a by-product of palm oil extraction. Besides, the residual palm kernel meal obtained from SFE process using CO_2_ can be a good, low fiber animal feed. Although the technique is struggling with the cost-effectiveness factors in the case of low-volume products, it is overcoming this limitation day by day and promising economic benefits. Based on this review, it can be concluded that SFE is the best method for exploitation in the extraction of palm oil on an industrial scale.
